# A Promising Small Molecule for Vanishing White Matter Disease

**DOI:** 10.15844/pedneurbriefs-32-5

**Published:** 2018-08-28

**Authors:** Divakar S. Mithal, Jennifer P. Rubin

**Affiliations:** 1 Division of Neurology, Department of Pediatrics, Ann and Robert H. Lurie Children’s Hospital of Chicago, Chicago, IL

**Keywords:** Integrated Stress Response, Vanishing White Matter Disease, Chemical Biology, eIF2B, Translation Initiation

## Abstract

Investigators from Calico Life Sciences LLC and AbbVie report the effects of a novel drug targeting the genetic basis of Vanishing White Matter Disease (VWMD).

Investigators from Calico Life Sciences LLC and AbbVie report the effects of a novel drug targeting the genetic basis of Vanishing White Matter Disease (VWMD). VWMD is caused by homozygous or compound heterozygous variants in any of five genes (eIF2B1, eIF2B2, eIF2B3, eIF2B4 or eIF2B5). Collectively the five genes encode a portion of the eukaryotic translation inhibition factor 2 (eIF2) termed eIF2B that is required for normal protein translation. VWMD-causing variants are though to cause a partial reduction in eIF2B activity. Prior studies by members of the group from Calico Life Sciences had shown that ISR-inhibitor (ISRIB), a small molecule, was able to activate and stabilize eIF2B [1]. The current investigation by Wong et al. demonstrates that ISRIB activates and stabilizes VWMD-specific eIF2B variants. [2]

COMMENTARY. VWMD is a chronic, episodic and progressive leukoencephalopathy with highly variable presentation. Features include essentially normal early life development followed by progressive ataxia, cognitive impairment, and spasticity. In 1997 Van Der Knapp et al., proposed four criteria for diagnosis (1) essentially normal initial motor and cognitive development, (2) chronic, progressive deterioration that often follows a mild infection or head trauma, (3) progressive cerebellar ataxia and spasticity are prominent features; epilepsy and optic atrophy may be present; cognitive impairments are less significant than motor impairments and (4) MRI findings are symmetric, with white matter signal intensity similar to cerebrospinal fluid on multiple sequences [3]. The disease can present at any age, and is usually fatal. Despite advances in diagnosis and management, treatments remain symptomatic and ultimately palliative.

When clinical suspicion is high, the diagnosis of VWMD is confirmed by genetic testing for homozygous or compound heterozygous variants in genes encoding the five eIF2B subunits. Normal protein expression requires function of eIF2B to extend translated RNA sequences [4]. While the translational function of eIF2B is critical to all cell types, VWMD is the only known disease caused by mutations in this complex.

The other important function of eIF2B is regulation of the integrated stress response (ISR), a reprogramming of protein expression that is common for a variety of cellular stress signals. ISR both shuts down normal protein expression, and activates specific transcription factors to help protect a stressed cell. Prior work demonstrated that suppression of eIF2B activates the ISR. ISRIB efficiently activates and stabilizes eIF2B to block the ISR [1].

VWMD is caused by diminished eIF2B activity and often worsens in the setting of illness, which may be related to improper regulation of the ISR. The current investigation examined whether ISRIB can utilize the hypoactive eIF2B found in VWMD to properly inhibit the ISR. The authors selectively edited the eIF2B gene with CRISPR-Cas9 in otherwise normal cells to recreate patient derived mutations. Cells were then treated with ISRIB. Consistent with a properly inhibited ISR, global protein expression was increased and ISR-specific transcription factors were decreased [2]. While the exact mechanisms of VWMD remain unclear, dysfunction of eIF2B remains the central disease-causing factor. Thus, the presented findings suggest that ISRIB may stabilize an abnormal eIF2B pathway in VWMD. At present, however, ISRIB has not been shown to change the course of VWMD in any disease models. When and how to use this medication is not evident from the current work but will need further investigation to establish pre-clinical efficacy.

## Disclosures

The author(s) have declared that no competing interests exist.
